# Reconstruction of Rift Valley fever transmission dynamics in Madagascar: estimation of force of infection from seroprevalence surveys using Bayesian modelling

**DOI:** 10.1038/srep39870

**Published:** 2017-01-04

**Authors:** Marie-Marie Olive, Vladimir Grosbois, Annelise Tran, Lalaina Arivony Nomenjanahary, Mihaja Rakotoarinoro, Soa-Fy Andriamandimby, Christophe Rogier, Jean-Michel Heraud, Veronique Chevalier

**Affiliations:** 1 CIRAD, Animal and Integrated Risk Management (AGIRs) Unit, Montpellier, France; 2 Institut Pasteur de Madagascar, Virology Unit, Antananarivo, Madagascar; 3 Institut Pasteur de Madagascar, Direction, Madagascar; 4 Institute for Biomedical Research of the French Armed Forces (IRBA), Brétigny-Sur-Orge, France; 5 Unité de recherche sur les maladies infectieuses et tropicales émergentes (URMITE), Marseille, France

## Abstract

The force of infection (FOI) is one of the key parameters describing the dynamics of transmission of vector-borne diseases. Following the occurrence of two major outbreaks of Rift Valley fever (RVF) in Madagascar in 1990–91 and 2008–09, recent studies suggest that the pattern of RVF virus (RVFV) transmission differed among the four main eco-regions (East, Highlands, North-West and South-West). Using Bayesian hierarchical models fitted to serological data from cattle of known age collected during two surveys (2008 and 2014), we estimated RVF FOI and described its variations over time and space in Madagascar. We show that the patterns of RVFV transmission strongly differed among the eco-regions. In the North-West and Highlands regions, these patterns were synchronous with a high intensity in mid-2007/mid-2008. In the East and South-West, the peaks of transmission were later, between mid-2008 and mid-2010. In the warm and humid northwestern eco-region favorable to mosquito populations, RVFV is probably transmitted all year-long at low-level during inter-epizootic period allowing its maintenance and being regularly introduced in the Highlands through ruminant trade. The RVF surveillance of animals of the northwestern region could be used as an early warning indicator of an increased risk of RVF outbreak in Madagascar.

Understanding the dynamics of transmission of infectious diseases is crucial for assessing disease risk and proposing adapted prevention and control measures^1^. Mathematical modeling is an approach frequently used to understand such dynamics and simulate control strategies such as vaccination^2^,^3^. However, the reliability of mathematical models depends upon the estimation of key parameters^2^. The force of infection (FOI), i.e. the probability of a susceptible individual to get infected over a time period, is one of these epidemiological parameters^1^,^2^,^4^,^5^. The FOI can be difficult to estimate directly through observation, monitoring or notification data for which reliability is strongly dependent upon the performances of surveillance systems^6^,^7^,^8^. An alternative approach is to estimate the FOI indirectly, through seroprevalence data collected on individuals of known age^1^,^4^, as already proposed for vector-borne diseases such as dengue and chikungunya^7^,^9^,^10^. Since the link between the data and the FOI to be estimated, is usually complex and implies a series of hierarchical relationships originated from multiple sources of dataset, using Bayesian hierarchical models is relevant^1^.

Rift Valley fever virus (RVFV) is an arthropod-borne zoonotic virus affecting mainly ruminants and human, that severely impacts the health and the economy in Africa, Arabian Peninsula and Indian Ocean, including Madagascar.

RVFV was isolated for the first time in Madagascar in 1979 from pools of mosquitoes collected in a glade of the Perinet forest where cattle stayed, within the Eastern margin of the central highlands, apart from any reported outbreak event^11^. This large island located in the Indian Ocean and characterized by a great diversity of eco-climatic patterns, was latter affected by two major Rift Valley fever (RVF) outbreaks in 1990–91 and 2008–09^12^,^13^,^14^,^15^. Although RVFV inter-epidemic transmission was suspected in 1995^16^ the pattern of RVFV circulation between epidemics is uncertain. Moreover, while retrospective investigations suggested that RVFV had been circulating among livestock since December 2007, the first RVF reported case was from a human in January 2008^12^. Then, outbreaks occurred in two epidemic waves during the two consecutive rainy seasons of 2008 and 2009^12^. The last RVF case was reported in March 2009 but recurrent circulation of the virus has since been detected in mid-2009, 2010–11 and 2012 on the Malagasy Highlands^17^,^18^,^19^. Since few data are available on RVF circulation during the 1990–91 and 2008–09 outbreaks as well as during the inter-epizootic period (IEP), the dynamics of transmission of RVF in Madagascar and the epidemiological mechanisms underlying these dynamics remain unclear. Additionally, following the 2008–09 outbreaks, it has been shown that there were considerable differences in the spatial distribution of RVF in both cattle and human^12^,^20^. Even though most of the RVF outbreaks were reported on the Highlands, a recent study suggested that some areas –i.e. the western and northwestern parts, were favorable to enzootic dynamics whereas others were suitable for epizootic dynamics^21^. Such differences indicated that the RVF dynamic patterns vary among Malagasy regions. In a wide country with limited resources as Madagascar, implementing a surveillance system accounting for the spatio-temporal RVF dynamic would optimize the strategies to better prevent and/or control the disease as well as the associated costs. Therefore, the objectives of our study were to estimate RVF FOI in Madagascar between 1992 and 2014, using cattle seroprevalence data and Bayesian hierarchical models, and to describe its variations over space and time.

## Material and Methods

### Serological surveys

**Figure 1:**
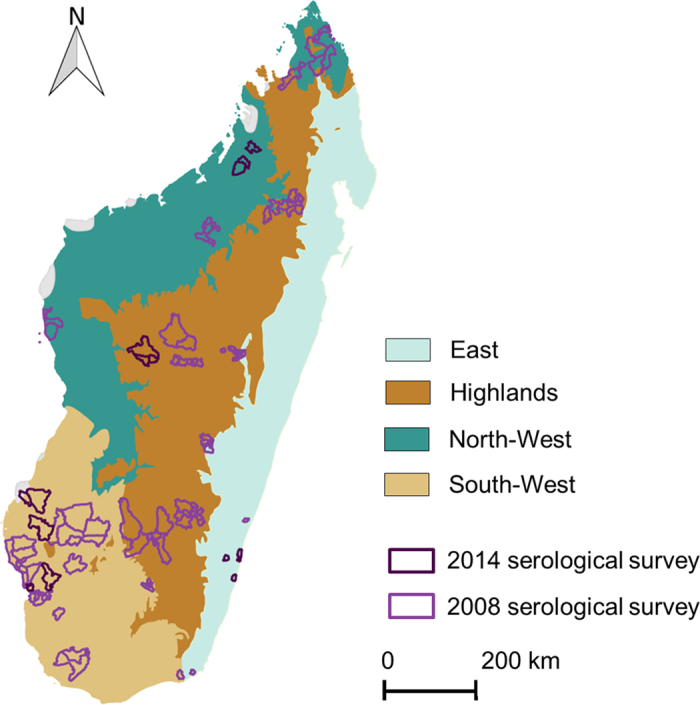
Communes sampled in the Malagasy eco-regions. This map was created using the software Quantum GIS version 1.8.0 (http://www.qgis.org/fr/site/).

Two serological surveys of cattle of known age were used to estimate the FOI in Madagascar. The first one was a published national cross-sectional survey conducted in August 2008 on 3,450 ruminants^20^ (Fig. 1). Only cattle which breeding location was known were considered in the present study (n = 1,432). Animal sera were analyzed using commercial ELISA kits (BDSL) to detect anti-RVFV immunoglobulin (Ig) G^22^. The sensitivity and specificity of the serological test for bovines were estimated to be 96.3% and 99.7% respectively^22^.

The second serosurvey was undertaken from March to May 2014 (n = 1,140; Fig. 1) to investigate the circulation of RVFV following the 2008–09 epidemics in Madagascar. Animals born after, during and before the outbreaks were therefore purposively sampled in the four main eco-regions of Madagascar defined by Cornet^13^. Two districts were considered in the South-West eco-region and one district in each of the three others eco-regions, namely the East, the Highlands and the North-West (Fig. 1). The sample was stratified according to age so that it included cattle with contrasted histories with regard to exposure to RVFV. In each district, at least 30 animals born after the last 2009 epizootic for each age categories (1, 2, 3 and 4 years) were sampled. We also sampled per district, at least 100 cattle that were more than 4 years old (born during or before the 2008–09 epizootic). Cattle sera were analyzed using a commercial ELISA kit (ID Screen Rift Valley Fever Competition Multispecies ELISA^®^) to detect antibody directed against RVFV. According to the ring trial performed by Kortekaas *et al*.^23^, we computed the mean sensitivity and specificity values of the test as 97.2% and 100% respectively.

Considering that the samplings were performed around the middle of the 2008 and middle of 2014, our annual estimations overlapped two years. We thus considered that our annual estimations of the FOI started from the middle of the year to the middle of the following year (e.g. mid-2002/mid-2003). Moreover, because the oldest animals in our sample were born in 1992 and the last sampling was done at the beginning of 2014, the time period for FOI estimation was 1992 to 2014.

### Estimation of the force of infection

Both 2008 and 2014 age stratified datasets were merged and used to estimate the annual FOI, based on the principle that age of individuals is an indicator of the cumulative time of potential infection^9^. Thus, depending on the year of birth and the year of sampling of each animal, its exposure to RVFV over each year from mid-1992 to mid-2014 was determined. It was assumed that once infected an individual has a lifelong sero-positivity against RVFV^24^ and that the mortality related to RVF infection was null.

The status *S*_*yal*_ according to the serological test of an individual sampled in year *y* at age *a* in locality *l* was considered as a random variable distributed according to a Bernouilli law of parameter *pa*_*yal*_ (Equation 1).


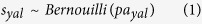


The probability of a positive test result, *pa*_*yal*_, was then related to the probability of an individual being seropositive *pv*_*yal*_ and to the sensitivity (*Se*_*i*_) and specificity (*Sp*_*i*_) of the serological tests used (Equation 2)


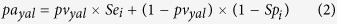


For any individual, *pv*_*yal*_ was considered as the complement of the probability of being seronegative at the year of sampling *y* and thus the complement of the probability of never having been infected from the year of birth *y-a* to the year of sampling *y*. This last probability is the product of the probabilities for a susceptible individual of not getting infected over each year from its year of birth *y-a* to the year of sampling *y*. Each of these probabilities is the complement of an annual force of infection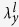 (the probability of a susceptible individual to get infected over a year *y* in locality *l*). The above reasoning can then be translated into the equation 3.


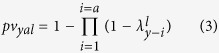


Finally, 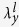 was considered as a random variable distributed according a Beta law (probability distribution defined on the interval [0, 1]) of parameters α and β (Equation 4).


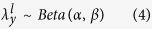


Several candidate models differing with respect to how 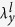 varied over space and time were fitted to the data.

### Scenarios

The study area was divided into four main regions, following a classification suggested by Cornet^13^ and the terrestrial major habitat types proposed by the World Wide Fund (WWF; http://wwf.panda.org/about_our_earth/ecoregions/ecoregion_list/; Fig. 1). These four eco-regions were chosen because of their ecological characteristics and socio-cultural practices in terms of herd management^25^ assumed to influence the RVFV transmission dynamic:

– East, per-humid environment, agricultural area where livestock farming is occasional or opportunistic and the animals are used for agricultural work;

– Highlands, cold, humid environment with mixed farming where the breeders are also agricultural farmers. The animals are used for agricultural work, soil improvement and milk production;

– North-West, sub-humid environment and important livestock breeding area. The breeders are animals’ producers even if the animals are also used for agricultural work;

– South-West, semi-arid environment and important livestock breeding area. This region is considered as the exclusive animals’ producer area where herds are raised extensively.

**Figure 2:**
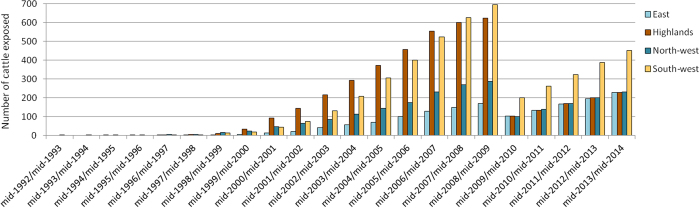
Number of sampled cattle exposed to RVF over each year from mid-1992 to mid-2014.

Since few of the sampled animals (less than 30) were exposed to the annual FOI from mid-1992 to mid-2002 (Fig. 2 and supplementary material 2), FOI was considered as constant and estimated for that period. On the other hand, the available samples allowed the estimation of an annual FOI from mid-2002/mid-2003 to mid-2013/mid-2014 (Fig. 2 and supplementary material 2).

Four scenarios were considered:

– Model 1: FOI did neither vary over space, nor over time (null model).

– Model 2: the FOI varied over the four eco-regions but not over time.

– Model 3: the FOI varied over time but not over space.

– Model 4: the FOI varied over the four eco-regions and over time.

For each model, the priors distributions assigned to the FOI parameters were uninformative beta distributions of parameters α = 1 and β = 1. The four multivariate Bayesian models were developed using OpenBUGS 3.2.3 (Medical Research Council Biostatistics Unit and Imperial College London, London, United Kingdom). Models were burned in for 10,000 iterations to achieve convergence, and estimates were then based on the next 90,000 iterations, thinning each ten iteration. The selection of the best model was based on the Deviance Information Criterion (DIC)^26^: lesser was the DIC better was the model.

More details about the Bayesian hierarchical models are provided in the supplementary material 1.

## Results

### Analysis of serological data

As a whole, 2,572 individual sera were considered for the analysis. A total of 1,432 individuals from the 2008 cattle dataset were included in the study (Table 1). Cattle age ranged from 1 to 12 years (median age 4 years, first quartile 3 years and third quartile 6 years). The overall seropositivity rate was 19.3% (Confident Interval (CoI) 95% [17.3–21.8]). In 2014, 1,140 individuals were sampled in 16 communes belonging to five Malagasy districts (Fig. 1 and Table 1). Cattle age ranged from 1 to 22 years (median age 4 years, first quartile 2 years and third quartile 6 years). The overall seropositivity rate was 7.9% (CoI 95% [6.4–9.6]). The seroprevalence rates were estimated to 7.9% (CoI 95% [4.7–12.2]), 6.1% (CoI 95% [3.4–10.0]), 9.1% (CoI 95% [5.7–13.6]), 8.2% (CoI 95% [5.8–11.1]) in the East, the Highlands, the North-West and South-West respectively. Seroprevalence rates were not statistically different between eco-regions (Chi square test).

From 2002 to 2014, more than 40 animals were exposed to RVFV per eco-regions and per year (Fig. 2 and supplementary material 2).

### Estimation of the force of infection

**Figure 3:**
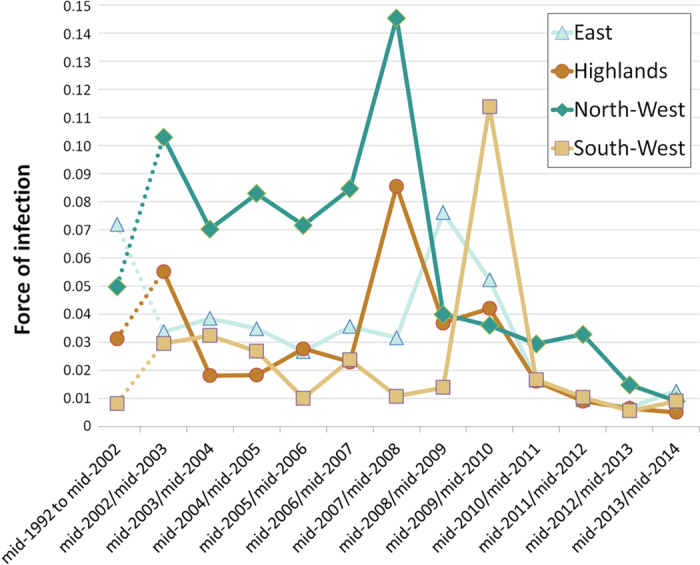
Estimated force of infection in four Malagasy eco-regions, mid-1992 to mid-2014, according to Model 4.

Model 4 in which the FOI varied over the four eco-regions and time was selected as the best model (Table 2). According to this model, the pattern of RVF transmission varied as follows (Table 2 and Fig. 3):

– In the North-West, the estimation of FOI was at 0.050 (95% Credible Interval (CI) [0.002–0.161]) from mid-1992 to mid-2002. In mid-2002/mid-2003 the FOI reached at 0.103 (95% CI [0.005–0.322]) and remained relatively high (0.07 95% CI [0.003–0.252] to 0.085 95% CI [0.005–0.226]) from mid-2003/mid-2004 to mid-2006/mid-2007. In mid-2007/mid-2008, the FOI peaked at 0.145 (95% CI [0.020–0.261]) and then decreased dramatically in mid-2008/mid-2009 at 0.040 (95% CI [0.001–0.164]) and then more slowly from 0.036 (95% CI [0.002–0.125]) in mid-2009/mid-2012 to 0.009 (95% CI [3.10–4–0.043]) in mid-2013/mid-2014;

– In the Highlands, the FOI was estimated at 0.031 (95% CI [0.002–0.089]) from mid-1992 to mid-2002. In mid-2002/mid-2003 the FOI was at 0.055 (95% CI [0.003–0.156]), decreased at 0.018 (95% CI [7.10–4–0.080]) in mid-2003/mid-2004 and remained between 0.018 (95% CI [7.10–4–0.076]) and 0.028 (95% CI [0.001–0.094]) until mid-2006/mid-2007. In mid-2007/mid-2008, the FOI increased strongly at 0.086 (95% CI [0.009–0.155]) and decreased between 0.037 (95% CI [0.002–0.121]) and 0.042 (95% CI [0.002–0.124]), in mid-2008/mid-2009 and mid-2009/mid-2010. Then, during the period from mid-2010/mid-2011 to mid-2013/mid-2014 the FOI was estimated between 0.005 (95% CI [2.10–4–0.027]) and 0.016 (95% CI [6.10–4–0.069]);

– In the East, the estimation of FOI was high (0.072 95% CI [0.004–0.234]) from mid-1992 to mid-2002. It remained at a lower level, between 0.027 (95% CI [0.001–0.113]) and 0.039 (95% CI [0.002–0.160]) from mid-2002/mid-2003 to mid-2007/mid-2008. The FOI then reached 0.076 (95% [0.006–0.171]) in mid-2008/mid-2009 and decreased to 0.052 (95% CI [0.003–0.153]) in mid-2009/mid-2010. Finally, during the mid-2010/mid-2011-mid-2013/mid-2014 period the FOI ranged between 0.007 (95% CI [2.10–4–0.035]) and 0.016 (95% CI [6.10–4–0.076]);

– In the South-West, the estimation of FOI was between 0.008 (95% CI [3.10–4–0.040]) and 0.032 (95% CI [0.002–0.098]) from mid-1992 to mid-2009. The FOI peaked at 0.114 (95% CI [0.036–0.186]) in mid-2009/mid-2010 and then decreased dramatically in mid-2010/mid-2011 and remained low until mid-2013/mid-2014 (between 0.006 95% CI [2.10–4–0.027] and 0.017 (95% CI [6.10–4–0.075]).

## Discussion

Estimation of the FOI have been derived from data on seroprevalence of known age individuals for several infectious diseases including measles, chikungunya and dengue^1^,^7^,^9^,^10^, but to our knowledge this methodology has never been used to estimate the FOI of RVF. In our study, the link between the data collected (*i.e.* results of serological tests) and the parameters to be estimated (*i.e.* forces of infection) was complex and implied a series of hierarchical relationships: the outcome of a serological test was related to the true serological status of the tested individual and the sensitivity and specificity of the serological test used; the true serological status of an individual was in turn related to the set of annual FOIs experienced over its lifetime. Such a complex structure could not be accounted for with frequentist statistics. In such a situation where, in addition, multiple sources of data had to be incorporated, Bayesian hierarchical models were appropriate to estimate the FOIs^27^. Using probability distributions for prior and posterior parameters permits to reflect the uncertainty in their values.

At first glance, the variation of RVF FOIs estimated from mid-1992 to mid-2014 is in accordance with the historical report of RVF transmission in Madagascar: high transmission during the 2008–09 outbreaks and low transmission outside this period^12^,^16^,^17^,^18^,^19^,^20^,^21^. Then, our results are consistent with the hypothesis of contrasted patterns of long-term RVFV enzootic circulation in the different Malagasy eco-regions. Indeed, in the northwestern part, the FOI was the highest during IEP as well as in mid-2007/mid-2008, probably before the 2008–09 outbreaks. In the other regions, the Highlands, the East and the South-West, the FOI was lower during the IEP with a peak of transmission between mid-2007 and mi-2010. The pattern of transmission in the North-West indicated that RVFV is probably transmitted all year long at moderate levels during IEP. This region is characterized by a humid and hot environment with large surface of waterbodies (artificial and natural) and high cattle density. Such an environment is favorable to the presence of RVF mosquito vectors throughout the year^28^, thus to an IEP maintenance of RVFV. The estimated FOI increased in mid-2007/mid-2008, suggesting that 2008–09 epidemics may have started by an intensification of RVFV transmission starting mid-2007 in the North-West and probably also in the Highlands. Indeed, while the FOI estimated in the central Highlands was globally lower than in the North-West, temporal variation in FOI seemed to be synchronous in these two regions. Indeed, as observed in the North-West, FOI in the Highlands suddenly increased in mid-2007/mid-2008 and then decreased in mid-2008/mid-2009 and mid-2009/mid-2010. This pattern is somewhat surprising, since most of the human and ruminant cases in the Highlands were recorded in 2008–09. However unreported outbreaks could also have occurred in 2007, before the first notifications^12^. The central Highlands are characterized, during the dry season, by a cold environment unfavorable for RVF vectors^13^,^29^,^30^. Yet, recurrent RVFV circulation has been detected in cattle in mid-2009, 2010–2011 and in human in 2012 in this region^17^,^18^,^19^. Finally, our estimations showed an unexpected FOI peak in mid-2002/mid-2003 in the northwestern and the Highlands regions. To our knowledge, no RVF outbreaks were reported at this time in Madagascar. In 2002, a political crisis occurred in the country, weakening the health system^31^,^32^, and because of this instability, outbreaks could have remained unnoticed.

Two hypotheses could explain the synchronous transmission pattern in northwestern and Highlands regions. Firstly, if existing, the climatic drivers of RVF circulation in both regions could be similar, allowing the RVFV enzootic maintenance and initiating outbreak emergence under specific conditions (*e.g.* heavy rainfall). Secondly, the regular and intense ruminant movements^33^,^34^ from the northwestern part towards the central Highlands markets and slaughterhouses could be responsible for continuous introduction of RVFV in the Highlands. An increased transmission in the northwestern region would lead to the introduction of viremic animals in the central Highlands and because of the low level of immunity of ruminants in this region, trigger outbreaks.

In the East of Madagascar, where the first outbreaks were recorded in 1990^15^, the FOI was high during the mid-1992 to mid-2002 period. Such a high transmission level could be explained by a persistent circulation favored by a hot and wet climate, following the 1990–91 epizootics. Then the FOI decreased between mid-2002 and mid-2007 and a peak of transmission was observed in mid-2008/mid-2009. This region is a humid environment favorable to vectors of RVFV but because of the low ruminant densities, the enzootic transmission could be limited to certain areas and outbreaks could be occasional and confined.

Finally, in the South-West of Madagascar the FOI was low except in mid-2009/mid-2010 when FOI suddenly increased. The southwestern part of Madagascar is a semi-arid region with few permanent and temporary waterbodies^21^. This environment is unfavorable to RVF mosquito vectors during the dry season^28^. However, our results indicated that, as in the Highlands, RVFV circulated at low level in this region, suggesting that the virus could have nonetheless been maintained.

To date, in Madagascar, drivers associated with RVFV enzootic maintenance and 2008–09 outbreaks are still poorly understood. Several mechanisms are likely involved in the persistence of RVFV: vertical transmission in mosquitoes, the existence of wild mammal reservoir populations, the maintenance of low level transmission associated with ruminant movements^24^,^35^,^36^. While the existence of wild terrestrial mammals as reservoir of RVF in Madagascar is unlikely^37^, the vertical transmission has still to be considered. Indeed, vertical transmission has been described in Aedes subgenus *Neomelaniconion* mosquitoes^38^ which is present in all Malagasy eco-regions^29^,^39^. The role of ruminant movements –barter and trade, in RVFV circulation and persistence, has already been shown at a local scale in a pilot area in the Highlands^19^,^33^,^40^. A better understanding of these movements all over the island, using field records and mathematical modeling is essential to assess their role in RVF epidemiology in Madagascar^40^. Regarding to outbreaks, Anyamba *et al*.^41^ used data on environmental drivers of RVF derived from satellite measurements (sea surface temperatures, rainfall and Normalized Difference Vegetation Index anomalies) to produce a RVF risk map for Madagascar. Only 23% of the RVF human cases reported in the South, North and Highlands of Madagascar in 2008–09 were located in high risk areas of this map which were mostly restricted to the northeastern part of Madagascar^41^,^42^. Our study highlights intense RVFV circulation in the North-West and Highlands of Madagascar in mid-2007/mid-2008 which further supports the hypothesis that RVF risk area is not restricted to the northeastern part of Madagascar. The time scale used in the study of Anyamba *et al*.^41^,^42^ may have not been optimal to detect relationship between RVF circulation and the climatic anomalies that initiated the intensification of RVF circulation. The flexibility of Bayesian hierarchical models would allow further expanding and improving our model through the incorporation of an additional layer in which the FOI would be related, through logit linear relationships, to environmental and climatic variables that were not taken into account in this work. This model could be used to better understand the mechanisms of emergence responsible for the increased FOI from 2007 to 2009 in Madagascar, opening the way towards predictive approach.

Although no data were available on the estimation of RVF FOI in the literature, the FOI may be compared to the rate of seroconversion estimated during longitudinal surveys^10^. Nicolas *et al*.^19^ estimated a seroconversion rate of 7% and 14% in 2009–2010 and 2010–2011 respectively in the Highlands of Madagascar. Our post-epizootic FOI estimation on the Highlands was lower (1.6% and 0.9%) than these estimations. Such a discrepancy is likely to result from space scale sampling differences: our estimation was done at the eco-regional level whereas the other one was performed in a district highly affected by RVF in 2008^12^,^19^. Two studies performed in the tropical islands of the South-West Indian Ocean showed an annual RVFV antibody acquisition about 17.5% in Union of Comoros in 2010–11^43^ and an IgM prevalence about 4% in 2010–11 in Mayotte^44^, which were higher than our estimations. Yet, the climatic situations in the Union of Comoros and Mayotte, under marine tropical conditions, are extremely different than in Madagascar. Furthermore, since 2002, importation of live animals from Tanzania is frequent in the Union of Comoros and could then increase the risk of RVFV circulation^43^. In the semi-arid area of the Barkedji region of Senegal, studies showed a seroconversion rate among monitored small ruminants of 1.9% between of 1991–93 and 2.9% during the rainy season of 2003^45^,^46^. These results are slightly higher than our estimation in the semi-arid region of the South-West during the enzootic period (around 1%) but this difference can be explained by the difference of susceptibility to RVFV between small ruminants and cattle^47^.

Limitations in our analyses may have affected our results. First we assumed that RVF infection does not cause an excess of mortality amongst cattle. Considering the moderate mortality rate among adult cattle (usually less than 10%)^47^ and the seven million of cattle in Madagascar, the misestimating due to this assumption would be low. Secondly, since our estimation of the FOI has been calculated from geographically limited sampling and extrapolated to Malagasy eco-region, the representativeness of our estimations by eco-regions has still to be cautiously considered. Thirdly, because few sampled animals were exposed to the annual FOI from mid-1992 to mid-2002, we considered a constant FOI for that period which may be wrong. However, ignoring the samples from animals born before 2002 would result in reduced statistical power and precision in parameter estimations. We therefore chose to keep those individuals in our analysis and considered a constant FOI from mid-1992 to mid-2002. Finally, the increase in FOI before the outbreak in the northwestern and Highlands parts was a single event. Long term monitoring may confirm or not, our estimations of RVF patterns of transmission.

## Conclusion

Using Bayesian hierarchical models fitted to cattle serological data, we showed that RVF transmission dynamics varied according to the eco-regions of Madagascar. Firstly, the variation of RVF FOI estimated from mi-1992 to mid-2014 seems in accordance with the historical report of RVF transmission in Madagascar. Secondly, our results suggest that the northwestern part of the island is an at-risk region for RVF enzootic transmission and that RVF transmission intensity increased there before the outbreaks, as well as in the Highlands. Consequently, the surveillance of RVF circulation in these regions would be an appropriate early warning tool. Additionally, as ruminant trade from the northwestern towards to the Highlands is known, the introduction of RVFV from the North-West part of Madagascar would be plausible. If this hypothesis is confirmed and considering that RVF history in Madagascar showed that Highlands are susceptible to RVF outbreaks, the RVF surveillance of animals coming to the Highlands’ markets and slaughterhouse from the northwestern region could also be used as an early warning method.

In conclusion, in order to ward off difficulties encountered by RVF case notification bias and uncertainties on RVF IEP circulation, we believe that the methodology used here is well appropriate to study RVF transmission dynamics, but also to estimate other epidemiological key parameters (mortality, morbidity), the financial cost of an outbreak and the assessment of the disease burden.

## Additional Information

**How to cite this article**: Olive, M.-M. *et al*. Reconstruction of Rift Valley fever transmission dynamics in Madagascar: estimation of force of infection from seroprevalence surveys using Bayesian modelling. *Sci. Rep.* **7**, 39870; doi: 10.1038/srep39870 (2017).

**Publisher's note:** Springer Nature remains neutral with regard to jurisdictional claims in published maps and institutional affiliations.

## Supplementary Material

Supplementary Material 1

Supplementary Material 2

## Figures and Tables

**Table 1:** Positive animals to anti-RVFV antibody and total number of cattle sampled in 2008 and 2014 in each Malagasy eco-region.

Eco-region	2008		2014		Total eco-region
	Positive	Total 2008	Positive	Total 2014	
East	21	99	18	228	327
Highlands	117	556	14	230	786
North-West	93	238	21	231	469
South-West	46	539	38	451	990
Total	277	1,432	91	1,140	2,572

**Table 2:** Comparison of Deviance Information Criterion (DIC) values and estimation of the Force of infection (FOI) and their 95% Credible Interval (CI) for each model (Model 1, Model 2, Model 3 and Model 4) from mid-1992 to mid-2014.

Model	Description	Period and region for FOI estimation	Median FOI	CI 95%	DIC
Model 1	No variation of the FOI over space and time	Madagascar	0.029	[0.027–0.033]	2018
Model 2	FOI varied over the four eco-regions but not over time	East	0.029	[0.021–0.039]	1961
		Highlands	0.040	[0.034–0.047]	
		North-West	0.066	[0.055–0.079]	
		South-West	0.022	[0.018–0.027]	
Model 3	FOI varied over time but not over space	mid-1992 to mid-2002	0.040	[0.006–0.080]	1921
		mid-2002/mid-2003	0.049	[0.003–0.126]	
		mid-2003/mid-2004	0.023	[0.001–0.081]	
		mid-2004/mid-2005	0.026	[0.001–0.077]	
		mid-2005/mid-2006	0.016	[7.10–4–0.057]	
		mid-2006/mid-2007	0.026	[0.001–0.074]	
		mid-2007/mid-2008	0.045	[0.003–0.109]	
		mid-2008/mid-2009	0.068	[0.010–0.120]	
		mid-2009/mid-2010	0.069	[0.023–0.114]	
		mid-2010/mid-2011	0.013	[6.10–4–0.046]	
		mid-2011/mid-2012	0.010	[5.10–4–0.031]	
		mid-2012/mid-2013	0.003	[1.10–4–0.015]	
		mid-2013/mid-2014	0.007	[0.002–0.018]	
Model 4	FOI varied over the four eco-regions and over time	East mid-1992 to mid-2002	0.050	[0.002–0.161]	1854
		mid-2002/mid-2003	0.103	[0.005–0.322]	
		mid-2003/mid-2004	0.070	[0.003–0.252]	
		mid-2004/mid-2005	0.083	[0.004–0.256]	
		mid-2005/mid-2006	0.072	[0.003–0.225]	
		mid-2006/mid-2007	0.085	[0.005–0.226]	
		mid-2007/mid-2008	0.145	[0.020–0.261]	
		mid-2008/mid-2009	0.040	[0.001–0.164]	
		mid-2009/mid-2010	0.036	[0.002–0.125]	
		mid-2010/mid-2011	0.029	[0.001–0.102]	
		mid-2011/mid-2012	0.033	[0.002–0.098]	
		mid-2012/mid-2013	0.015	[5.10–4–0.062]	
		mid-2013/mid-2014	0.009	[3.10–4–0.043]	
		Highlands mid-1992 to mid-2002	0.031	[0.002–0.089]	
		mid-2002/mid-2003	0.055	[0.003–0.156]	
		mid-2003/mid-2004	0.018	[7.10–4–0.080]	
		mid-2004/mid-2005	0.018	[7.10–4–0.076]	
		mid-2005/mid-2006	0.028	[0.001–0.094]	
		mid-2006/mid-2007	0.023	[9.10–4–0.085]	
		mid-2007/mid-2008	0.086	[0.009–0.155]	
		mid-2008/mid-2009	0.037	[0.002–0.121]	
		mid-2009/mid-2010	0.042	[0.002–0.124]	
		mid-2010/mid-2011	0.016	[6.10–4–0.069]	
		mid-2011/mid-2012	0.009	[3.10–4–0.045]	
		mid-2012/mid-2013	0.007	[2.10–4–0.033]	
		mid-2013/mid-2014	0.005	[2.10–4–0.027]	
		North-West mid-1992 to mid-2002	0.072	[0.004–0.234]	
		mid-2002/mid-2003	0.034	[0.001–0.159]	
		mid-2003/mid-2004	0.039	[0.002–0.160]	
		mid-2004/mid-2005	0.035	[0.001–0.147]	
		mid-2005/mid-2006	0.027	[0.001–0.113]	
		mid-2006/mid-2007	0.036	[0.002–0.132]	
		mid-2007/mid-2008	0.032	[0.001–0.119]	
		mid-2008/mid-2009	0.076	[0.006–0.171]	
		mid-2009/mid-2010	0.052	[0.003–0.153]	
		mid-2010/mid-2011	0.016	[6.10–4–0.076]	
		mid-2011/mid-2012	0.010	[3.10–4–0.048]	
		mid-2012/mid-2013	0.007	[2.10–4–0.035]	
		mid-2013/mid-2014	0.013	[0.002–0.040]	
		South-West mid-1992 to mid-2002	0.008	[3.10–4–0.040]	
		mid-2002/mid-2003	0.030	[0.001–0.103]	
		mid-2003/mid-2004	0.032	[0.002–0.098]	
		mid-2004/mid-2005	0.027	[0.001–0.081]	
		mid-2005/mid-2006	0.010	[4.10–4–0.044]	
		mid-2006/mid-2007	0.024	[0.001–0.063]	
		mid-2007/mid-2008	0.011	[4.10–4–0.043]	
		mid-2008/mid-2009	0.014	[5.10–4–0.050]	
		mid-2009/mid-2010	0.114	[0.036–0.186]	
		mid-2010/mid-2011	0.017	[6.10–4–0.075]	
		mid-2011/mid-2012	0.010	[4.10–4–0.044]	
		mid-2012/mid-2013	0.006	[2.10–4–0.027]	
		mid-2013/mid-2014	0.009	[0.001–0.028]	
