# Supplementary material for: Reconstruction of Rift Valley fever transmission dynamics in Madagascar: estimation of force of infection from seroprevalence surveys using Bayesian modelling
Source: Sci Rep. 2017 Jan 4;7:39870. doi: 10.1038/srep39870 (PMC5209714; doi:10.1038/srep39870)
Supplement: Supplementary Material 1 [file srep39870-s1.pdf]

Reconstruction of Rift Valley fever transmission dynamics in Madagascar: estimation of force of infection from seroprevalence surveys using Bayesian modelling

Marie-Marie Olive, Vladimir Grosbois, Annelise Tran, Lalaina Arivony Nomenjanahary, Mihaja Rakotoarinoro, Soa-Fy Andriamandimby, Christophe Rogier, Jean-Michel Heraud, Veronique Chevalier

Supplementary material 1 : Bayesian hierarchical models

Organization of the data

For each animal, depending on the year of birth and the year of sampling, its exposure to RVFV over each year from mid-1992 to mid-2014 was determined as following:

1. for the period of mid-1992 to mid-2002, for each animal we considered the number of years of exposure;
2. for the other years, if the animal was present during the year considered, its exposure was 1 and 0 if not.

Two examples are provided in the table below:

1. an animal of 8 years old sampled mid-2008, was considered born in mid-2000. We thus considered that it was exposed 2 years during the period of mid-1992/mid-2002 (mid-2000/mid-2001 and mid-2001/mid-2002), and exposed each year from mid-2002/mid-2003 to mid-2007/mid-2008 but not exposed from mid-2008/mid-2009 to mid-2013/mid-2014.
2. an animal of 5 years old sampled in mid-2014, was considered born in mid-2009. It was not exposed to RVFV before mid-2009, and then each year from mid-2009/mid-2010 to mid-2013/mid-2014.

DOB	DOS	mid-1992 to mid-2002	mid-2002/ mid-2003	mid-2003/ mid-2004	mid-2004/ mid-2005	mid-2005/ mid-2006	mid-2006/ mid-2007	mid-2007/ mid-2008	mid-2008/ mid-2009	mid-2009/ mid-2010	mid-2010/ mid-2011	mid-2011/ mid-2012	mid-2012/ mid-2013	mid-2013/ mid-2014
Mid-2000	Mid-2008	2	1	1	1	1	1	1	0	0	0	0	0	0
Mid-2009	Mid-2014	0	0	0	0	0	0	0	0	1	1	1	1	1
...	...	...	...	...	...	...	...	...	...	...	...	...	...	...

DOB : Date of birth

DOS: Date of sampling

Model

The status s_{yal} according to the serological test of an individual sampled in year *y* at age *a* in locality *l* was considered as a random variable distributed according to a Bernouilli law of parameter pa_{yal} (Equation 1).

$$s_{yal} \sim Bernouilli(pa_{yal}) \quad (1)$$

The probability of a positive test result, pa_{yal}, was then related to the probability of an individual being seropositive pv_{yal} and to the sensitivity (Se_i) and specificity (Sp_i) of the serological tests used (Dohoo et al., 2012; Equation 2)

$$pa_{yal} = pv_{yal} \times Se_i + (1 - pv_{yal}) \times (1 - Sp_i) \quad (2)$$

For any individual, pv_{yal} was considered as the complement of the probability of being seronegative at the year of sampling *y* and thus the complement of the probability of never having been infected from the year of birth *y-a* to the year of sampling *y*. This last probability is the product of the probabilities for a susceptible individual of not getting infected over each year from its year of birth *y-a* to the year of sampling *y*. Each of these probabilities is the complement of an annual force of infection λ_y^l (the probability of a susceptible individual to

get infected over a year y in locality l). The above reasoning can then be translated into the equation 3.

$$pv_{yal} = 1 - \prod_{i=1}^{i=a}\left(1 - \lambda_{y-i}^{l}\right) \quad (3)$$

Finally, λ_y^l was considered as a random variable distributed according a Beta law (probability distribution defined on the interval [0, 1]) of parameters α and β (Equation4).

$$\lambda_y^l \sim Beta(\alpha, \beta) \quad (4)$$

For each model, the priors distributions assigned to the FOI parameters were uninformative beta distributions of parameters α=1 and β=1.

Script on OpenBugs

Acronyms

binary variables indicating exposure for each year

p92_02 = mid-1992 to mid-2002

p02 = mid-2002/mid-2003

p03 = mid-2003/mid-2004

p04 = mid-2004/mid-2005

p05= mid-2005/mid-2006

p06= mid-2006/mid-2007

p07= mid-2007/mid-2008

p08= mid-2008/mid-2009

p09= mid-2009/mid-2010

p10= mid-2010/mid-2011

p11= mid-2011/mid-2012

p12= mid-2012/mid-2013

p13= mid-2013/mid-2014

pa = probability of a positive test result

pv = probability of being seropositive

Se = sensibility of the serological test

Sp = specificity of the serological test

Force of infections (λ)

l92_02e = FOI mid-1992 to mid-2002 in the east region

l02e = FOI mid-2002/mid-2003 in the east region

l03e = FOI mid-2003/mid-2004 in the east region

l04e = FOI mid-2004/mid-2005 in the east region

l05e = FOI mid-2005/mid-2006 in the east region

l06e = FOI mid-2006/mid-2007 in the east region

l07e = FOI mid-2007/mid-2008 in the east region

l08e = FOI mid-2008/mid-2009 in the east region

l09e = FOI mid-2009/mid-2010 in the east region

l10e = FOI mid-2010/mid-2011 in the east region

l11e = FOI mid-2011/mid-2012 in the east region

l12e = FOI mid-2012/mid-2013 in the east region

l13e = FOI mid-2013/mid-2014 in the east region

l92_02h = FOI mid-1992 to mid-2002 in the highlands

l02h = FOI mid-2002/mid-2003 in the highlands

l03h = FOI mid-2003/mid-2004 in the highlands

l04h = FOI mid-2004/mid-2005 in the highlands

l05h = FOI mid-2005/mid-2006 in the highlands

l06h = FOI mid-2006/mid-2007 in the highlands

l07h = FOI mid-2007/mid-2008 in the highlands

l08h = FOI mid-2008/mid-2009 in the highlands

l09h = FOI mid-2009/mid-2010 in the highlands

l10h = FOI mid-2010/mid-2011 in the highlands

l11h = FOI mid-2011/mid-2012 in the highlands

l12h = FOI mid-2012/mid-2013 in the highlands

l13h = FOI mid-2013/mid-2014 in the highlands

lp92_02n = FOI mid-1992 to mid-2002 in the north-west region

l02n = FOI mid-2002/mid-2003 in the north-west region

l03n = FOI mid-2003/mid-2004 in the north-west region

l04n = FOI mid-2004/mid-2005 in the north-west region

l05n = FOI mid-2005/mid-2006 in the north-west region

l06n = FOI mid-2006/mid-2007 in the north-west region

l07n = FOI mid-2007/mid-2008 in the north-west region

l08n = FOI mid-2008/mid-2009 in the north-west region

l09n = FOI mid-2009/mid-2010 in the north-west region

l10n = FOI mid-2010/mid-2011 in the north-west region

l11n = FOI mid-2011/mid-2012 in the north-west region

l12n = FOI mid-2012/mid-2013 in the north-west region

l13n = FOI mid-2013/mid-2014 in the north-west region

lp92_02s = FOI mid-1992 to mid-2002 in the south-west region

l02s = FOI mid-2002/mid-2003 in the south-west region

l03s = FOI mid-2003/mid-2004 in the south-west region

l04s = FOI mid-2004/mid-2005 in the south-west region

l05s = FOI mid-2005/mid-2006 in the south-west region

l06s = FOI mid-2006/mid-2007 in the south-west region

l07s = FOI mid-2007/mid-2008 in the south-west region

l08s = FOI mid-2008/mid-2009 in the south-west region

l09s = FOI mid-2009/mid-2010 in the south-west region

l10s = FOI mid-2010/mid-2011 in the south-west region

l11s = FOI mid-2011/mid-2012 in the south-west region

l12s = FOI mid-2012/mid-2013 in the south-west region

l13s = FOI mid-2013/mid-2014 in the south-west region

Model 1: FOI did neither vary over space, nor over time (null model).

According to this model, considering that the FOI was constant over years, the probability of being seronegative is the probability of not getting infected over a year (complement of the annual force of infection λ) at the power age of the animal.

$$pv_{yal} = 1 - [(\mathbf{1} - \boldsymbol{\lambda})^{age}]$$

```
model
{
for (i in 1:2572)
{
Y[i]~dbin(pa[i],1)
```

```
pa[i]<-pv[i]*Se[i]+(1-pv[i])*(1-Sp[i])
pv[i]<-1-pow((1-l),age[i])


#step Se
#if test=1 then Se=Se1=97.2% else Se=Se2=93.3%
#step Sp
#if test=1 then Sp=Sp1=100% else Sp=Sp2=100%


Se[i]<-(step(test[i]-1)*0.972)+(1-step(test[i]-1))*(0.933)
Sp[i]<-(step(test[i]-1)*1)+(1-step(test[i]-1))*(1)


}
l~dbeta(1,1)


 }
```

Model 2: the FOI varied over the four eco-regions but not over time.

This model differs slightly from the Model 1, because the force of infection varies across regions but not over years.

```
model
{
## East ##
for (i in 1:327)
{
Y[i]~dbin(pa[i],1)
```

```
pa[i]<-pv[i]*Se[i]+(1-pv[i])*(1-Sp[i])

pv[i]<-1-pow((1-l1),age[i])


#step Se

#if test=1 then Se=Se1=97.2% else Se=Se2=93.3%

#step Sp

#if test=1 then Sp=Sp1=100% else Sp=Sp2=100%


Se[i]<-(step(test[i]-1)*0.972)+(1-step(test[i]-1))*(0.933)

Sp[i]<-(step(test[i]-1)*1)+(1-step(test[i]-1))*(1)

}


##Highlands ##

for (j in 328:1113)

{

Y[j]~dbin(pa[j],1)

pa[j]<-pv[j]*Se[j]+(1-pv[j])*(1-Sp[j])

pv[j]<-1-pow((1-l2),age[j])


#step Se

#if test=1 then Se=Se1=97.2% else Se=Se2=93.3%

#step Sp

#if test=1 then Sp=Sp1=100% else Sp=Sp2=100%


Se[j]<-(step(test[j]-1)*0.972)+(1-step(test[j]-1))*(0.933)
```

```
Sp[j]<-(step(test[j]-1)*1)+(1-step(test[j]-1))*(1)

}



##North-West##

for (k in 1114:1582)

{

Y[k]~dbin(pa[k],1)

pa[k]<-pv[k]*Se[k]+(1-pv[k])*(1-Sp[k])

pv[k]<-1-pow((1-l3),age[k])


#step Se

#if test=1 then Se=Se1=97.2% else Se=Se2=93.3%

#step Sp

#if test=1 then Sp=Sp1=100% else Sp=Sp2=100%


Se[k]<-(step(test[k]-1)*0.972)+(1-step(test[k]-1))*(0.933)

Sp[k]<-(step(test[k]-1)*1)+(1-step(test[k]-1))*(1)

}


##South-West##

for (m in 1583:2572)

{

Y[m]~dbin(pa[m],1)

pa[m]<-pv[m]*Se[m]+(1-pv[m])*(1-Sp[m])
```

```
pv[m]<-1-pow((1-l4),age[m])


#step Se
#if test=1 then Se=Se1=97.2% else Se=Se2=93.3%
#step Sp
#if test=1 then Sp=Sp1=100% else Sp=Sp2=100%


Se[m]<-(step(test[m]-1)*0.972)+(1-step(test[m]-1))*(0.933)
Sp[m]<-(step(test[m]-1)*1)+(1-step(test[m]-1))*(1)
}



#Priors lambda
l1~dbeta(1,1)
l2~dbeta(1,1)
l3~dbeta(1,1)
l4~dbeta(1,1)
}
```

Model 3: the FOI varied over time but not over space.

In this model the FOI is different according to the year considered but does not differ between the region. Let us for example, consider a 5 years old individual sampled in mid-2014 (so born in mid-2009). Its probability of being seronegative is the product of the

probabilities of not getting infected in mid-2009/mid-2010, in mid-2010/mid-2011, in mid-2011/mid-2012, in mid-2012/mid-2013 and in mid-2013/mid-2014.

$$pv_{yal} = 1 - \left[\left(\mathbf{1} - \lambda^{l}_{mid-2013/mid-2014}\right) * \left(\mathbf{1} - \lambda^{l}_{mid-2012/mid-2013}\right) * \left(\mathbf{1} - \lambda^{l}_{mid-2011/mid-2012}\right) * \left(\mathbf{1} - \lambda^{l}_{mid-2010/mid-2011}\right) * \left(\mathbf{1} - \lambda^{l}_{mid-2009/mid-2010}\right)\right]$$

```
model
{
for (i in 1:2572)
{
Y[i]~dbin(pa[i],1)
pa[i]<-pv[i]*Se[i]+(1-pv[i])*(1-Sp[i])
pv[i]<-1-(pow((1-l92_02), p92_02[i])*pow((1-l02),p02[i])* pow((1-l03),p03[i])*
pow((1-l04),p04[i])* pow((1-l05),p05[i])* pow((1-l06),p06[i])* pow((1-l07),p07[i])*
pow((1-l08),p08[i])* pow((1-l09),p09[i])* pow((1-l10),p10[i])* pow((1-l11),p11[i])*
pow((1-l12),p12[i])* pow((1-l13),p13[i]))

#step Se
#if test=1 then Se=Se1=97.2% else Se=Se2=93.3%
#step Sp
#if test=1 then Sp=Sp1=100% else Sp=Sp2=100%

Se[i]<-(step(test[i])*0.972)+(1-step(test[i]))*(0.963)
Sp[i]<-(step(test[i])*1)+(1-step(test[i]))*(0.997)


}
```

```
l92_02  ~dbeta(1,1)
l02~dbeta(1,1)
l03~dbeta(1,1)
l04~dbeta(1,1)
l05~dbeta(1,1)
l06~dbeta(1,1)
l07~dbeta(1,1)
l08~dbeta(1,1)
l09~dbeta(1,1)
l10~dbeta(1,1)
l11~dbeta(1,1)
l12~dbeta(1,1)
l13~dbeta(1,1)

    }
```

Model 4: the FOI varied over the four eco-regions and over time.

This model differs slightly from the Model 3, because the force of infection varies across regions as well as over years.

```
model
{

### East ###
```

```
for (i in 1:327)
{
Y[i]~dbin(pa[i],1)
pa[i]<-pv[i]*Se[i]+(1-pv[i])*(1-Sp[i])
pv[i]<-1-(pow((1-l92_02e), p92_02[i])*pow((1-l02e),p02[i])* pow((1-l03e),p03[i])*
pow((1-l04e),p04[i])* pow((1-l05e),p05[i])* pow((1-l06e),p06[i])* pow((1-
l07e),p07[i])* pow((1-l08e),p08[i])* pow((1-l09e),p09[i])* pow((1-l10e),p10[i])*
pow((1-l11e),p11[i])* pow((1-l12e),p12[i])* pow((1-l13e),p13[i]))

#step Se
#if test=1 then Se=Se1=97.2% else Se=Se2=96.3%
#step Sp
#if test=1 then Sp=Sp1=100% else Sp=Sp2=99.7%

Se[i]<-(step(test[i])*0.972)+(1-step(test[i]))*(0.963)
Sp[i]<-(step(test[i])*1)+(1-step(test[i]))*(0.997)

}

### Highlands ###

for (j in 328:1113)
{
Y[j]~dbin(pa[j],1)
```

```
pa[j]<-pv[j]*Se[j]+(1-pv[j])*(1-Sp[j])

pv[j]<-1-(pow((1-l92_02h), p92_02[j])*pow((1-l02h),p02[j])* pow((1-l03h),p03[j])*
pow((1-l04h),p04[j])* pow((1-l05h),p05[j])* pow((1-l06h),p06[j])* pow((1-
l07h),p07[j])* pow((1-l08h),p08[j])* pow((1-l09h),p09[j])* pow((1-l10h),p10[j])*
pow((1-l11h),p11[j])* pow((1-l12h),p12[j])* pow((1-l13h),p13[j]))

#step Se
#if test=1 then Se=Se1=97.2% else Se=Se2=96.3%
#step Sp
#if test=1 then Sp=Sp1=100% else Sp=Sp2=99.7%

Se[j]<-(step(test[j])*0.972)+(1-step(test[j]))*(0.963)
Sp[j]<-(step(test[j])*1)+(1-step(test[j]))*(0.997)

}

### clust3 = north-west = n ###

for (k in 1114:1582)
{
Y[k]~dbin(pa[k],1)
pa[k]<-pv[k]*Se[k]+(1-pv[k])*(1-Sp[k])
pv[k]<-1-(pow((1-l92_02n), p92_02[k])*pow((1-l02n),p02[k])* pow((1-
l03n),p03[k])* pow((1-l04n),p04[k])* pow((1-l05n),p05[k])* pow((1-l06n),p06[k])*
```

```
pow((1-l07n),p07[k])* pow((1-l08n),p08[k])* pow((1-l09n),p09[k])* pow((1-l10n),p10[k])* pow((1-l11n),p11[k])* pow((1-l12n),p12[k])* pow((1-l13n),p13[k]))

#step Se
#if test=1 then Se=Se1=97.2% else Se=Se2=96.3%
#step Sp
#if test=1 then Sp=Sp1=100% else Sp=Sp2=99.7%

Se[k]<-(step(test[k])*0.972)+(1-step(test[k]))*(0.963)
Sp[k]<-(step(test[k])*1)+(1-step(test[k]))*(0.997)

}

### clust4 = south-west = s ###

for (m in 1583:2572)
{
Y[m]~dbin(pa[m],1)
pa[m]<-pv[m]*Se[m]+(1-pv[m])*(1-Sp[m])
pv[m]<-1-(pow((1-l92_02s), p92_02[m])*pow((1-l02s),p02[m])* pow((1-l03s),p03[m])* pow((1-l04s),p04[m])* pow((1-l05s),p05[m])* pow((1-l06s),p06[m])* pow((1-l07s),p07[m])* pow((1-l08s),p08[m])* pow((1-l09s),p09[m])* pow((1-l10s),p10[m])* pow((1-l11s),p11[m])* pow((1-l12s),p12[m])* pow((1-l13s),p13[m]))

#step Se
```

```
#if test=1 then Se=Se1=97.2% else Se=Se2=96.3%

#step Sp

#if test=1 then Sp=Sp1=100% else Sp=Sp2=99.7%


Se[m]<-(step(test[m])*0.972)+(1-step(test[m]))*(0.963)

Sp[m]<-(step(test[m])*1)+(1-step(test[m]))*(0.997)


}



l92_02e~dbeta(1,1)

l02e~dbeta(1,1)

l03e~dbeta(1,1)

l04e~dbeta(1,1)

l05e~dbeta(1,1)

l06e~dbeta(1,1)

l07e~dbeta(1,1)

l08e~dbeta(1,1)

l09e~dbeta(1,1)

l10e~dbeta(1,1)

l11e~dbeta(1,1)

l12e~dbeta(1,1)

l13e~dbeta(1,1)


l92_02h ~dbeta(1,1)
```

l02h~dbeta(1,1)

l03h~dbeta(1,1)

l04h~dbeta(1,1)

l05h~dbeta(1,1)

l06h~dbeta(1,1)

l07h~dbeta(1,1)

l08h~dbeta(1,1)

l09h~dbeta(1,1)

l10h~dbeta(1,1)

l11h~dbeta(1,1)

l12h~dbeta(1,1)

l13h~dbeta(1,1)

l92_02n ~dbeta(1,1)

l02n~dbeta(1,1)

l03n~dbeta(1,1)

l04n~dbeta(1,1)

l05n~dbeta(1,1)

l06n~dbeta(1,1)

l07n~dbeta(1,1)

l08n~dbeta(1,1)

l09n~dbeta(1,1)

l10n~dbeta(1,1)

l11n~dbeta(1,1)

l12n~dbeta(1,1)

l13n~dbeta(1,1)

l92_02s ~dbeta(1,1)

l02s~dbeta(1,1)

l03s~dbeta(1,1)

l04s~dbeta(1,1)

l05s~dbeta(1,1)

l06s~dbeta(1,1)

l07s~dbeta(1,1)

l08s~dbeta(1,1)

l09s~dbeta(1,1)

l10s~dbeta(1,1)

l11s~dbeta(1,1)

l12s~dbeta(1,1)

l13s~dbeta(1,1)

}

Reference

Dohoo, I.R., Martin, W., Stryhn, H., in *Methods in epidemiologic research*. 96-130 (VER, 2012)
