# Supplementary material for: Reconstruction of Rift Valley fever transmission dynamics in Madagascar: estimation of force of infection from seroprevalence surveys using Bayesian modelling
Source: Sci Rep. 2017 Jan 4;7:39870. doi: 10.1038/srep39870 (PMC5209714; doi:10.1038/srep39870)
Supplement: Supplementary Material 2 [file srep39870-s2.pdf]

Reconstruction of Rift Valley fever transmission dynamics in Madagascar: estimation of force of infection from seroprevalence surveys using Bayesian modelling

Marie-Marie Olive, Vladimir Grosbois, Annelise Tran, Lalaina Arivony Nomenjanahary, Mihaja Rakotoarinoro, Soa-Fy Andriamandimby, Christophe Rogier, Jean-Michel Heraud, Veronique Chevalier

Supplementary material 2 : Number of individuals exposed to RVFV over each year from mid-1992 to mid-2014. For the period of mid-1992 to mid-2002, the number of animals exposed to RVFV were n=50, n=296, n=174, n=158 and n=678 for East, Highlands, North-West, South-West and overall Madagascar respectively.

	mid-1992/ mid-1993	mid-1993/ mid-1994	mid-1994/ mid-1995	mid-1995/ mid-1996	mid-1996/ mid-1997	mid-1997/ mid-1998	mid-1998/ mid-1999	mid-1999/ mid-2000	mid-2000/ mid-2001	mid-2001/ mid-2002	mid-2002/ mid-2003	mid-2003/ mid-2004	mid-2004/ mid-2005	mid-2005/ mid-2006	mid-2006/ mid-2007	mid-2007/ mid-2008	mid-2008/ mid-2009	mid-2009/ mid-2010	mid-2010/ mid-2011	mid-2011/ mid-2012	mid-2012/ mid-2013	mid-2013/ mid-2014
East	0	0	2	2	2	2	3	6	13	20	41	57	71	101	128	150	169	102	135	166	196	228
Highlands	0	0	0	0	4	7	12	35	94	144	215	292	373	457	554	600	623	103	134	169	200	230
North-West	2	2	2	2	6	6	17	24	47	66	85	114	144	174	232	271	287	101	138	169	200	231
South-West	0	0	0	0	2	3	14	18	45	76	132	209	305	400	523	627	695	200	262	324	387	451
Total	2	2	4	4	14	18	46	83	199	306	473	672	893	1132	1437	1648	1774	506	669	828	983	1140
